# Epiphytic bacterial community composition on two common submerged macrophytes in brackish water and freshwater

**DOI:** 10.1186/1471-2180-8-58

**Published:** 2008-04-10

**Authors:** Melanie Hempel, Maja Blume, Irmgard Blindow, Elisabeth M Gross

**Affiliations:** 1 Limnology, Department of Biology, University of Konstanz, PO Box 659, D-78457 Konstanz, Germany; 2 Biological Station of Hiddensee, University of Greifswald, D-18565 Kloster, Germany

## Abstract

**Background:**

Plants and their heterotrophic bacterial biofilm communities possibly strongly interact, especially in aquatic systems. We aimed to ascertain whether different macrophytes or their habitats determine bacterial community composition. We compared the composition of epiphytic bacteria on two common aquatic macrophytes, the macroalga *Chara aspera *Willd. and the angiosperm *Myriophyllum spicatum *L., in two habitats, freshwater (Lake Constance) and brackish water (Schaproder Bodden), using fluorescence *in situ *hybridization. The bacterial community composition was analysed based on habitat, plant species, and plant part.

**Results:**

The bacterial abundance was higher on plants from brackish water [5.3 × 10^7 ^cells (g dry mass)^-1^] than on plants from freshwater [1.3 × 10^7 ^cells (g dry mass)^-1^], with older shoots having a higher abundance. The organic content of freshwater plants was lower than that of brackish water plants (35 vs. 58%), and lower in *C. aspera *than in *M. spicatum *(41 vs. 52%). The content of nutrients, chlorophyll, total phenolic compounds, and anthocyanin differed in the plants and habitats. Especially the content of total phenolic compounds and anthocyanin was higher in *M. spicatum*, and in general higher in the freshwater than in the brackish water habitat. Members of the Cytophaga-Flavobacteria-Bacteroidetes group were abundant in all samples (5–35% of the total cell counts) and were especially dominant in *M. spicatum *samples. Alphaproteobacteria were the second major group (3–17% of the total cell counts). Betaproteobacteria, gammaproteobacteria, and actinomycetes were present in all samples (5 or 10% of the total cell counts). Planctomycetes were almost absent on *M. spicatum *in freshwater, but present on *C. aspera *in freshwater and on both plants in brackish water.

**Conclusion:**

Bacterial biofilm communities on the surface of aquatic plants might be influenced by the host plant and environmental factors. Distinct plant species, plant part and habitat specific differences in total cell counts and two bacterial groups (CFB, planctomycetes) support the combined impact of substrate (plant) and habitat on epiphytic bacterial community composition. The presence of polyphenols might explain the distinct bacterial community on freshwater *M. spicatum *compared to that of *M. spicatum *in brackish water and of *C. aspera *in both habitats.

## Background

In aquatic systems, bacteria occur often associated with surfaces, e.g. in biofilms or on lake or marine snow [1]. Biofilm associated bacteria are most abundant at intermediate nutrient availability while either low or high nutrient conditions favour planktonic growth of bacteria [2]. Biofilms are not only formed on abiotic surfaces but also on living organisms such as aquatic plants and algae.

In freshwater and marine habitats, bacteria associated with cyanobacterial blooms, diatom blooms, phytoplankton [3], lake snow [4], and bacterioplankton [5,6] have been investigated. Betaproteobacteria occur almost exclusively in freshwater but not in saline habitats, while alphaproteobacteria are more abundant in marine than in freshwater samples [5]. Alphaproteobacteria dominate the planktonic bacteria in the North Sea, followed by the Cytophaga-Flavobacteria-Bacteroidetes (CFB) group, and all groups of bacteria display a seasonal succession [7]. Diverse bacterial communities dominate in cyanobacterial blooms, including members of the CFB group and betaproteobacteria [8]. Mainly members of the CFB group and alphaproteobacteria, especially *Roseobacter*, are attached to marine diatoms [9,10]. Members of the CFB group and alpha-, beta-, and gammaproteobacteria have been identified by molecular methods on the chlorophytes *Desmidium devillii, Hyalothexca dissliens, *and *Spondylosium pulchrum *[11]. In general, the bacteria associated with diatoms and some chlorophytes that have been studied are mostly heterotrophic. In contrast, information about bacterial biofilms on aquatic macrophytes is scarce. A general overview and comparisons of attached and planktonic bacterial communities in freshwater and marine habitats is given in [12,13] and references therein.

Submerged macrophytes are, in addition to algae, the main primary producers in lakes; they structure the littoral zone and prevent resuspension of sediments, thus enabling clear water states [14]. The freshwater macrophytes *Myriophyllum spicatum *and *Chara globularis, *and possibly also other *Chara *species, produce secondary compounds such as polyphenols and cyclic sulfur compounds, which exert allelopathic activity against algae and cyanobacteria [15,16]. Antibacterial cyclic quaternary amines have been isolated from *C. globularis *[17]. Hydrolysable polyphenols of *M. spicatum*, especially tellimagrandin II, inhibit photosystem II of cyanobacteria [18]. Plant polyphenols may have antimicrobial activity, but some bacteria may also overcome polyphenol-based plant defences [19].

Not only secondary metabolites but also nutrients possibly affect biofilm density and composition. Depending on their life cycle stage, macrophytes may release low to substantial amounts of macronutrients [20], and at times high concentrations of micronutrients [21]. Especially older plant parts may leak both organic compounds and inorganic nutrients [22]. Nutrient conditions affect the impact of submerged macrophytes on bacterioplankton: *Vallisneria americana *has a positive impact on bacterioplankton density under high NH_4_^+ ^conditions, but a neutral or negative impact when NH_4_^+ ^is limiting [23].

Biofilms can be both beneficial and detrimental for submerged macrophytes. On the positive side, epiphytic biofilms provide organic compounds and carbon dioxide to the macrophytes and enhance nutrient recycling [24]. Further, the biofilm bacteria *Roseobacter gallaciencis *and *Pseudoalteromonas tunicata *that colonize the marine alga *Ulva australis *produce compounds against fouling organisms [25], and axenic *Ulva linza *require bacteria to restore the typical growth form, and some bacteria even enhance the algal growth rate [26,27]. Negative impacts on submerged macrophytes could arise from increased shading by thick biofilms and possibly also from pathogenic bacteria present in the biofilm. Macroalgae can also have negative effects on epiphytic bacteria. For instance, bacterial colonization of the marine red algae *Bonnemaisonia hamifera *and *Delisea pulchra *is inhibited by algal-released secondary metabolites [28,29]. These furanones also affect the swarming motility of *Serratia liquefaciens *[30] and indirectly affect larval attachment [31]. Whether or not such chemical interactions between plants and bacteria are important for biofilm density and community composition on aquatic macrophytes is unknown. The only study addressing microbial diversity on *M. spicatum *showed that the biofilm was dominated by gammaproteobacteria and members of the CFB group [32]. Bacterial epiphytes of *C. aspera *have not been described before.

Given that a strong interaction might exist between plants and their associated heterotrophic biofilm, especially in aquatic systems, we questioned whether different macrophytes (substrate, plant age) or the respective habitat determines bacterial community composition. We selected two common, allelochemically active, submerged macrophytes, *Chara aspera *and *Myriophyllum spicatum*, sampled in freshwater (Lake Constance) and brackish water (Schaproder Bodden). We identified plant species, plant age, and habitat-specific differences and similarities of the bacterial density and community composition.

## Results

**Figure 1:**
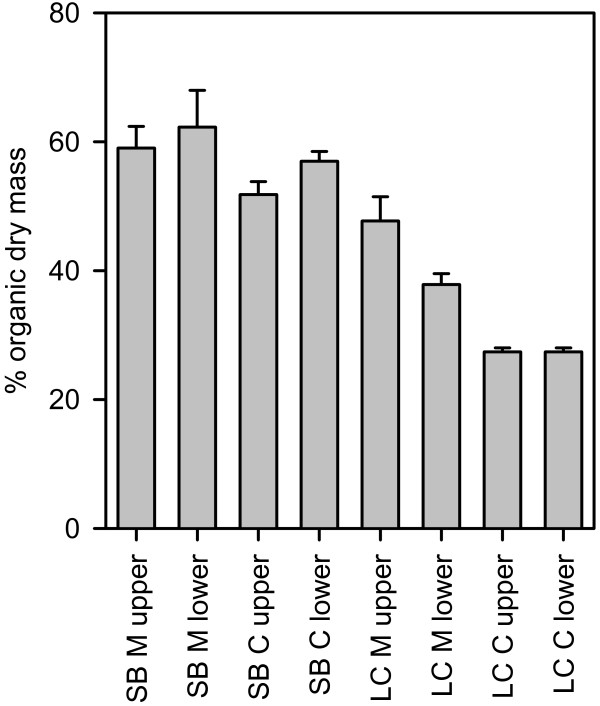
**Proportion of organic dry mass in plant samples collected at all sites**. SB: Schaproder Bodden, LC: Lake Constance, C: *Chara aspera*, M: *Myriophyllum spicatum; *upper and lower indicate plant parts analysed; n = 3; error bars indicate SE.

The two plant species, each from two different habitats, exhibited distinct morphological and chemical characteristics. The organic content of the plants of each species and from each habitat differed, but the upper or lower parts of each plant sampled did not differ in organic content (Figure 1; 3-way ANOVA, Table 1). *Chara aspera *had a lower organic content (40.9% ± 14.4, mean ± SD, n = 12) than *Myriophyllum spicatum *(51.7% ± 11.6, mean ± SD, n = 12). Freshwater plants had a much lower organic content (35.1% ± 9.4, mean ± SD, n = 12) than brackish water plants (57.5% ± 6.6, mean ± SD, n = 12). Only a marginal interaction of plant × habitat was found (p = 0.079), owing to a larger difference in organic content of *C. aspera *from the two sites than that of *M. spicatum*. The significant interaction term between habitat and plant part is due to the observed differences of plant parts in Lake Constance; the organic content of the plant parts did not differ in plants from Schaproder Bodden.

**Table 1:** Statistical analysis

		**% Plant organic matter**		**Ash-free dry mass**		**Total bacterial cell counts**		**Planctomycetes**		**CFB**	
						
**Source of variation**	**DF**	**F**	**P**	**F**	**P**	**F**	**P**	**F**	**P**	**F**	**P**
**Habitat**	1	101.63	**<0.001**	13.721	**0.002**	25.963	**<0.001**	30.970	**<0.001**	0.467	0.504
**Plant**	1	24.481	**<0.001**	3.746	*0.071*	1.944	0.182	26.623	**<0.001**	45.454	**<0.001**
**Plant part (PP)**	1	0.0563	0.815	0.183	0.674	21.229	**<0.001**	3.705	*0.072*	21.018	**<0.001**
**Habitat × PP**	1	3.510	*0.079*	0.0307	0.863	2.606	0.126	10.618	**0.005**	0.538	0.474
**Habitat × Plant**	1	5.249	**0.036**	2.253	0.153	10.499	**0.005**	0.484	0.497	4.901	**0.042**
**Plant × PP**	1	1.087	0.313	0.0479	0.830	0.0246	0.877	0.0998	0.756	7.105	**0.017**
**Habitat × Plant × PP**	1	0.505	0.488	0.121	0.732	< 0.001	0.995	1.179	0.294	14.113	**0.002**

3-way ANOVA for selected parameters. Data for the CFB were arcsin transformed; for planctomycetes, x^1/4 ^transformation was used.

*Myriophyllum spicatum *contained more phenolic compounds than *C. aspera *[97–173 mg (g dry mass)^-1 ^vs. <1 mg (g dry mass)^-1^; Table 2] and *M. spicatum *from Lake Constance had a slightly higher polyphenol content than *M. spicatum *from Schaproder Bodden [apices: 173 ± 21 mg (g dry mass)^-1 ^and 120 ± 33 mg (g dry mass)^-1^, respectively; Student's *t*-test: P = 0.02]. Also the anthocyanin content was much higher in *M. spicatum *than in *C. aspera*. In both habitats, the anthocyanin content of *C. aspera *was <0.1 mg (g dry mass)^-1^; the anthocyanin content of *M. spicatum *from Schaproder Bodden was slightly lower than that of *M. spicatum *from Lake Constance [approx. 0.5 mg (g dry mass)^-1 ^vs. 1.0 mg (g dry mass)^-1^; Student's *t*-test: P = 0.005, Table 1]. The chlorophyll *a *and *b *contents were highest in the apical shoots and upper leaves of *M. spicatum *from Lake Constance (Table 2).

**Table 2:** Chemical parameters measured in plants

	**Total phenolic content** **[mg (g dry mass)^-1^]**		**Anthocyanin** **[mg (g dry mass)^-1^]**		**Chlorophyll *a *and *b*** **[mg (g dry mass)^-1^]**	
			
	**LC**	**SB**	**LC**	**SB**	**LC**	**SB**
***C. aspera *** ***M. spicatum***	0.9 ± 0.08	0.7 ± 0.12	0.05 ± 0	0.06 ± 0	1.9 ± 0.17	1.2 ± 0.02
**Apex**	173 ± 21	120 ± 33	0.9 ± 0.02	0.52 ± 0.08	6.8 ± 1.3	2.3 ± 0.26
**Upper leaves**	120 ± 29	97 ± 5	0.8 ± 0.2	0.50 ± 0.01	8.4 ± 2.3	2.3 ± 0.28
**Upper stem**	133 ± 13	100 ± 9	1.4 ± 0.1	0.81 ± 0.15	1.8 ± 0.6	1.0 ± 0.04
						
	**C** **[mg (g dry mass)^-1^]**		**N** **[mg (g dry mass)^-1^]**		**P** **[mg (g dry mass)^-1^]**	
			
	**LC**	**SB**	**LC**	**SB**	**LC**	**SB**

***C. aspera *** ***M. spicatum***	206 ± 4	179 ± 15	11 ± 1.1	14 ± 0.12	0.4 ± 0.1	0.68 ± 0.06
**Apex**	425 ± 20	357 ± 35	36 ± 9	24 ± 8	2.6 ± 1.2	2.4 ± 1.5
**Upper leaves**	384 ± 37	363 ± 5	27 ± 6	16 ± 3	1.2	1.3 ± 0.33
**Upper stem**	400 ± 7	379 ± 25	15 ± 3	14 ± 3	0.9 ± 0.2	--

LC: Lake Constance, SB: Schaproder Bodden, n = 3, mean ± SD

The carbon content (Table 2) of *C. aspera *was about half of that of *M. spicatum*, possibly in part owing to the overall lower organic dry mass of the former. *Chara aspera *also contained less nitrogen and phosphorus per g dry mass than *M. spicatum *when whole plants were considered. The C/N molar ratio ranged from about 15 in apices of *M. spicatum *fromLake Constance to 31 in stems of *M. spicatum *from Schaproder Bodden. The C/P molar ratio ranged from 436 in apices of *M. spicatum *to more than 1373 in *C. aspera *from Lake Constance.

**Figure 2:**
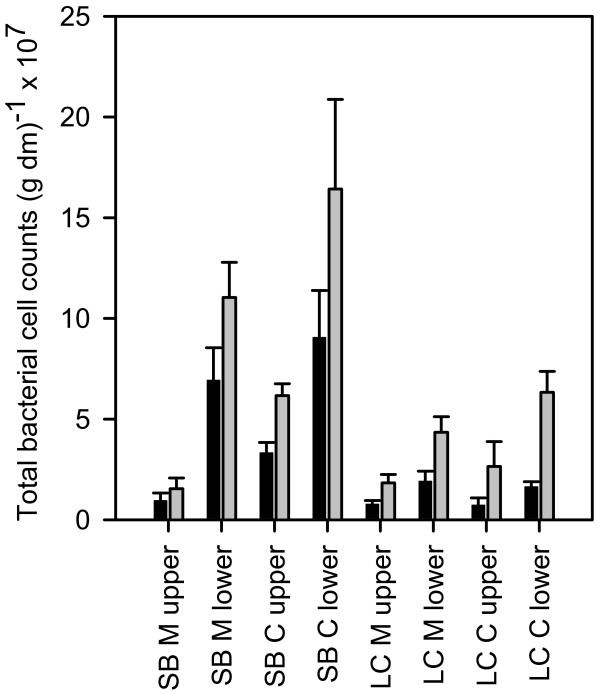
**Total bacterial cell counts determined by DAPI staining**. Black bars: counts (g dry mass)^-1^; grey bars: counts (g ash-free dry mass)^-1^. SB: Schaproder Bodden; LC: Lake Constance; M: *M. spicatum*; C: *C. aspera*. n = 3; error bars indicate SE.

We determined the bacterial abundance based on plant dry mass since there are no reliable surface area-to-biomass ratios for *M. spicatum *and *C. aspera *from the two habitats. The bacterial abundance in the two habitats and on the different plant parts differed significantly, but did not differ significantly between the two plant species (Figure 2, Table 1). In general, we found higher a bacterial abundance on plants from Schaproder Bodden [5.1 × 10^7 ^± 3.9 × 10^7 ^cells (g dry mass)^-1^; mean ± 1 SD] than on plants from Lake Constance [1.3 × 10^7 ^± 0.7 × 10^7 ^cells (g dry mass)^-1^]. The lower plants parts from Schaproder Bodden had higher bacterial cell counts than the upper plant parts, while cell counts on lower plant parts from Lake Constance were only marginally higher than the counts on upper plant parts (Figure 2), resulting in a significant habitat × plant part interaction (Table 1, P = 0.005). The ash-free dry mass differed significantly between habitats, and the organic content of the plant samples differed significantly between habitats and plant species but not between plant parts (Table 1). The general pattern of bacterial abundance remained when calculated on an organic dry matter basis (Figure 2).

**Figure 3:**
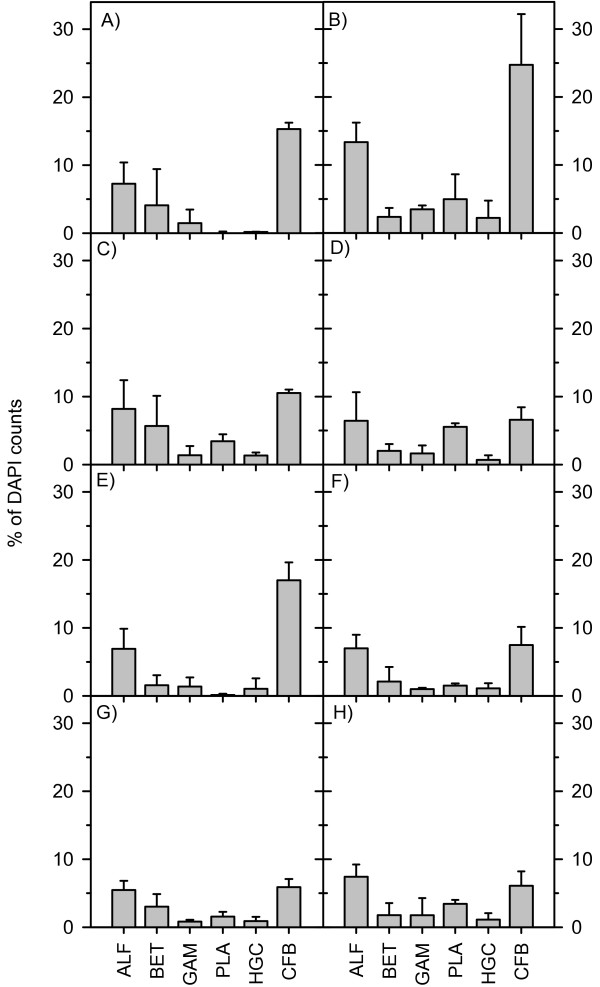
**Biofilm composition in Lake Constance (left) and Schaproder Bodden (right)**. A and B, *M. spicatum *upper section; C and D, *C. aspera *upper section; E and F, *M. spicatum *lower section; G and H, *C. aspera *lower section. n = 3; errors bars indicate SD. ALF: alphaproteobacteria; BET: betaproteobacteria; GAM: gammaproteobacteria; PLA: planctomycetes; HGC: actinomycetes; CFB: Cytophaga-Flavobacteria-Bacteroidetes.

The composition of the bacterial biofilm on the two plant species was similar except for the abundance of members of the CFB group and planctomycetes (Figure 3). On both plant species in both habitats, bacteria of the CFB group were the most abundant bacterial group and reached up to 35% of the total cell counts. The CFB counts correlated positively with all measured chemical parameters (Pearson correlation: carbon: r = 0.637, P = 0.0008; nitrogen: r = 0.666, P = 0.0003; phosphorus: r = 0.755, P < 0.0001; chlorophyll: r = 0.433, P = 0.0344; total phenolic compounds: r = 0.685, P = 0.0002). The number of CFB cells was generally higher on *M. spicatum *than on *C. aspera *and higher on upper parts of both plant species. The differences were not uniform and resulted in significant interaction terms (Figure 3; Table 1), which indicated specific habitat, plant, and plant part patterns.

The second major group of bacteria in the biofilms were alphaproteobacteria, which accounted for 3–17% of the DAPI counts. The abundance of alphaproteobacteria did not differ between plant species and habitats (3-way ANOVA, df = 1, F = 4.1, P = 0.05). Beta- and gammaproteobacteria abundance was similar on both plant species and in both habitats (3-way ANOVA, df = 1, F = 1.257, P = 0.279; df = 1, F = 1.982, P = 0.178). Actinomycetes were the least-abundant group, and their abundance did not differ between plant species (0.7–2.0% of DAPI counts, 3-way ANOVA, df = 1, F = 1.179, P = 0.294).

Interestingly, the proportion of planctomycetes differed between habitat and plant species. In Lake Constance, almost no planctomycetes were detected on *M. spicatum*, but they made up 2–3% of all cell counts on *C. aspera*. In Schaproder Bodden, planctomycetes were found on both plant species, with slightly higher numbers on the upper plant parts (2–6% of DAPI counts) than on the lower plant parts, but there were no differences between the plant species (Figure 3, Table 1). We found negative correlations of this group with carbon (Pearson correlation; r = -0.507, P = 0.0114), nitrogen (r = -0.433, P = 0.0343), chlorophyll (r = -0.648, P = 0.0006), and total phenolic content (r = -0.449, P = 0.0278).

**Figure 4:**
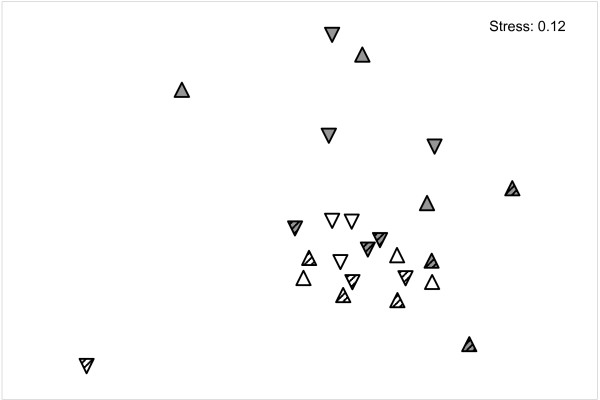
**Non-metric dimensional scaling plot of the bacterial community composition on all plant samples**. Grey triangles: *M. spicatum*, white triangles: *C. aspera*. Striped triangles: samples from Schaproder Bodden; non-striped triangles: samples from Lake Constance. Upper and lower plant parts are denoted by triangles pointing upwards and downwards, respectively. Data are x^1/4 ^transformed.

Overall, the bacterial community composition on *M. spicatum *in Lake Constance differed from that on *C. aspera *in both habitats and even from *M. spicatum *in Schaproder Bodden (Figure 4).

## Discussion

To our knowledge, this is the first study comparing bacterial biofilms on two macrophytes in brackish and freshwater habitats. Our data support the findings of other studies of biofilms on aquatic organisms, especially diatoms and cyanobacteria, where CFB and alphaproteobacteria make up major parts of the biofilm [8,10]. The total bacterial cell counts on the two plant species revealed that habitat and plant part seem to be more important for epiphyte bacterial abundance than the plant species. Although surface area-to-dry mass ratios have been determined in other studies, e.g. *Myriophyllum spicatum *1205 cm^2 ^(g dry mass)^-1 ^and *Nitellopsis obtusa *(starry stonewort) 560 cm^2 ^(g dry mass)^-1 ^[33], we decided not to calculate bacterial density based on plant surface area because our computer-based image analysis of *M. spicatum *leaf area showed that the calculation of surface area based on dry mass cannot be averaged over the whole plant. The surface area-to-dry mass ratio was 3500 cm^2 ^(g dry mass)^-1 ^for freshwater *M. spicatum *apices and 1600 cm^2 ^(g dry mass)^-1 ^for the lower parts of the same plant. Such a difference would amplify our findings that lower shoots harbour a higher abundance of bacteria. Freshwater *Chara *spp. had a surface area-to-biomass ratio of only 122 cm^2 ^(g dry mass)^-1^, which would yield even higher bacterial densities on this plant. In general, bacterial counts were highest on lower leaves close to the sediment (Figure 2). This seems reasonable since biofilm on older leaves should be thicker, thus containing more cells. Older leaves also contain less allelopathic compounds and are leakier than younger leaves, which might influence the nutrient availability. The nutrient content of the water column could be higher close to the sediment; this could also have an impact, but was not assessed in this study. Differences between the total bacterial cell counts on plant species from the different habitats might also be a consequence of pH, temperature, salinity and water retention time, which have been also found to influence community composition [34,35].

Alpha-, beta-, and gammaproteobacteria were present on both macrophytes in similar abundance, with gammaproteobacteria having the lowest counts of the proteobacteria. The least-abundant group was the actinomycetes (0.7–2.0% of DAPI counts). Not all members of this group might have been detected with the FISH probes because of the generally thicker cell walls of gram-positive bacteria. Our EUB probe, for example, detected only 50–80% of all DAPI cell counts (data not shown). The coverage of all bacteria together could probably have been higher if a combination of three different EUB probes were used [36], but since the planctomycetes, which are often missed by the single EUB probe used, did not make up a major amount of the biofilm, our results would not have changed dramatically. The total counts of all group-specific probes did not account for all eubacterial counts. We therefore assume that we did not detect all bacterial groups present in the biofilm of the two plant species, but we did use probes for the most common groups in biofilms and aquatic systems.

Alpha- and betaproteobacteria are the most abundant bacteria in lake snow aggregates in Lake Constance, and CFB are only found in hypolimnic particles, where they are considered to degrade refractory compounds such as chitin and cellulose [37]. Betaproteobacteria in the polluted river Spittelwasser dominated biofilms formed on glass slides throughout the year, followed by alphaproteobacteria, with seasonal maxima of CFB and planctomycetes, but gammaproteobacteria were never abundant [38]. Comparably, alphaproteobacteria, followed by CFB, were dominant in biofilms on stainless steel and polycarbonate exposed in Delaware Bay, and betaproteobacteria were almost absent [39]. In our study, we saw a comparable picture, with CFB mostly dominating the biofilm on macrophytes, followed by alpha- and betaproteobacteria. Distinct differences for habitat and plant species were found for the members of the CFB group and planctomycetes. Especially the abundance of planctomycetes differed between plant species and between the plant parts. Planctomycetes are found in a wide variety of habitats and are known to colonize surfaces [40]. Earlier studies on lake snow in Lake Constance did not look for this bacterial group, and a comparison of lakes and oceans found only low numbers in freshwater and hardly any in the marine bacterioplankton [5,37]. The authors of the latter study argue that this might be due to low abundance and that FISH was at the range of its detection limit. This could also be the case in our study since we only found low abundances. Based on our data and the meagre knowledge about planctomycetes ecology, we propose that either nutrient content or plant age (senescence) might account for differences in the abundance of planctomycetes because of the strong negative correlations with carbon, nitrogen, chlorophyll, and total phenolic content. This is supported by a study of marine planctomycetes, which were affected by organic compounds [41], and the observation that *M. spicatum *excretes substantial amounts of organic compounds [42]. Planctomycetes occur in many different habitats, yet their ecology is unexplored since only a few species have been cultivated [43].

The dominance of the CFB group in all our samples is not unusual, but is nevertheless interesting because we found distinct differences between plant species and plant parts. The CFB counts correlated positively with plant carbon and nutrient content as well as with chlorophyll and phenolic compounds. Members of the CFB group have often been described as major components of biofilms and are known to degrade rather complex molecules that occur in the high molecular mass fraction of dissolved organic matter [44]. This is important for other bacterial groups that are not capable of degrading such molecules but thrive on the degradation products [24]. Considering that alphaproteobacteria are more likely to degrade labile organic matter [37,45], this group could depend on degradation products of CFB or betaproteobacteria. The correlation of alphaproteobacteria with CFB (r = 0.444, P = 0.03) might be evidence for this.

We found that habitat had a distinct influence on both planctomycetes and members of the CFB group. The Schaproder Bodden is a shallow coastal area of the Baltic Sea and has a higher salinity than Lake Constance. Salinity is in fact a major environmental determinant of microbial community composition [35]. Whether or not the trophic state of the habitat influences biofilm density and composition remains open. Epiphytic algae are influenced by the trophic state, especially nitrogen availability, but only indirectly or not at all by host species [46]. Under eutrophic conditions, epiphytes should receive more organic and inorganic resources from the surrounding water and should be less dependent on plant-exuded compounds. It is unlikely that plant nutrient content influences the algal biofilm since plants relocate only small amounts of macronutrients [20]. This, however, does not exclude the possibility that the epiphytic algal composition might influence the composition of heterotrophic bacteria.

Only *M. spicatum *from Lake Constance exhibited a distinct bacterial biofilm community compared to *M. spicatum *from Schaproder Bodden and *C. aspera *from both habitats (Figure 4). Perhaps the phenolic content of the plant species in the different habitats is responsible for this effect. While *C. aspera *contains almost no phenolic compounds, *M. spicatum *has high concentrations of total phenolic compounds and anthocyanin, especially in the samples from Lake Constance.

We propose that the bacterial community composition is rather determined by the presence or (near) absence of phenolic compounds and not by their concentrations, since the concentrations between the upper and lower shoots in both habitats are rather similar. The association of polyphenol-degrading bacteria with *M. spicatum *[47] might be evidence for this. A direct proof of the impact of phenolic compounds on biofilm composition is difficult to achieve, and complex molecules, especially tannins, can be a difficult substrate for some bacteria [19] and may have caused this pattern. We also cannot rule out other factors such as salinity, pH, temperature, and dissolved organic carbon to explain these differences [48]. Dissolved organic matter produced by plants and epiphytic algae is usually subjected to photolysis [49] but can also be degraded by bacteria capable of degrading high molecular weight compounds [44]. The resulting degradation products as well as carbon dioxide and oxygen recycling can be of mutual benefit for both primary producers and heterotrophic bacteria. Biofilm bacteria are also known to produce compounds that can influence phototrophs beneficially or detrimentally [25]. Both plant species contain allelochemicals known to inhibit bacterial or algal growth [16,50].

## Conclusion

We present one of the first studies investigating heterotrophic bacterial communities on aquatic plants. To elucidate further which bacterial groups are active and contribute metabolically to processes within the biofilm, further approaches such as MICRO-FISH could be applied. To gain insight into the bacterial groups involved more specific probes for proteobacteria and CFB should be used. Also archaea could be of interest since they play a major role in the root region of various aquatic plants. Our data suggest an apparent impact of plant species, plant age and habitat on epiphytic bacterial communities.

## Methods

### Plants

Brackish water samples of *Chara aspera *and *Myriophyllum spicatum *were collected on 24 October 2006 in the Schaproder Bodden, east of the Isle of Hiddensee (N 54°27.4627'; E 13°07.5664'). Three plants each were collected by snorkelling in 0.7–1 m depth, stored in artificial brackish water (8‰, the same salinity as in the Bodden;[51]) with 3.5% formaldehyde (final concentration). Freshwater plants were sampled at the southwest shore of the Isle of Reichenau, Lake Constance, near a gravel ridge (N 47°42.247, E 9°02.289). Three replicates each were collected on 6 November 2006 at a depth of 0.7–1.2 m for *M. spicatum *and 2.5–3 m for *C. aspera*. The plants were transported in separate sterile tubes to the laboratory, where they were fixed with 3.5% formaldehyde (final concentration). All samples were stored at 4°C until processing started on 7 November 2006. The plant samples were divided into an upper section of the plants apices, approx. 5 cm long, and a lower section, approximately 5–10 cm of stem length above the sediment.

### Biomass and chemical analyses

*Myriophyllum spicatum *was processed as part of our routine sampling campaign, in which plants are dissected into apices, upper and lower leaves, and stems. For *C. aspera *chemical analyses, we did not differentiate between upper and lower plant parts. Sub-samples of each plant part were incinerated for 6 h at 550°C to determine the ash-free dry mass. We measured the carbon, nitrogen, and phosphorus content of all plant samples using standard methods [52]. The total phenolic content of *M. spicatum *and *C. aspera *was determined using a modified Folin-Ciocalteau method [53]. The concentration of non-phenolic compounds interfering with the Folin reagent are <5% in *M. spicatum *[52]. Those in *C. aspera *were determined using a modified polyvinylpyrrolidon method [50]. The major allelochemical of *M. spicatum*, tellimagrandin II, was quantified by reverse-phase HPLC [47]. All measurements were based on dry mass since the inorganic incrustations of *C. aspera *also provide settlement surfaces for bacteria. The antibacterial and allelopathically active compounds in *Chara *spp. [11,14] are difficult to isolate and were not determined here.

### Detachment of biofilm

Plant parts were transferred into sterile 50 ml polypropylene tubes containing 50 ml of formaldehyde (3.7% final concentration) and sodium pyrophosphate (0.1 M Na_4_P_2_O_7 _× 10 H_2_O, NaPPi). The biofilm was detached by ultrasonication for 60 s (Laboson 200 ultrasonic bath, Bender & Hobein), followed by 15 min of vigorous shaking (18.3 Hz, Thermomixer Eppendorf) and again 60 s of ultrasonication. Two millilitres of the detached biofilm were filtered onto white polycarbonate filters (0.2 μm, Ø 25 mm Nucleopore) and stored at -20 C.

We optimized the detachment procedure prior to this experiment. NaPPi was a suitable detergent to detach bacteria from macrophyte leaves as shown by a previous study in our group [47]. We further varied the sonication time and shaking duration to obtain the best results for a gentle but effective detachment of the biofilm [54,55]. Detachment with an ultrasonic probe (Bandelin electronic GM 70 HD, 20 kHz, 57W) resulted in 0.13 ± 0.03 × 10^6 ^cm^-2 ^but the plant tissue was severely damaged and numerous bacterial cells were still attached to the leaf surface as observed by microscopic examination. We then tried are more gentle detachment with shorter sonication times in an ultrasonic bath and constant, gentle shaking afterwards, rather than permanent ultrasonication. This method yielded 1.9 ± 0.6 × 10^6 ^cells cm^-2 ^and the plant tissue was not visibly damaged except at the cut surface on the petiole. A thorough microscopy of the leaves proved hardly any attached bacterial cells left.

### Fluorescence *in situ *hybridization (FISH)

FISH was conducted following a protocol by Pernthaler et al. [56] consisting of a hybridization step at 46°C for 3 h and a washing step for 15 min at 48°C. Filters were counterstained with DAPI (4',6-diamidino-2-phenylindol, 1 μg ml^-1^, 5 min). Stained cells were counted under an epifluorescence microscope (Labophot 2, Nikon) at an excitation wavelength of 549 nm. Probes used are listed in Table 3 and further details are available at probeBase [57].

**Table 3:** Oligonucleotide probes used in this study

**Probe^a)^**	**Sequence**	**% Formamide**	**Target group**	**Reference**
**EUB338**	GCTGCCTCCCGTAGGAGT	35	Most bacteria	[58]
**NON338**	ACTCCTACGGGAGGCAGC	35	Competitor of EUB	[59]
**ALF968**	GGTAAGGTTCTGCGCGT	20	Alphaproteobacteria	[60]
**BET42a^b)^**	GCCTTCCCACTTCGTTT	35	Betaproteobacteria	[61]
**GAM42a^b)^**	GCCTTCCCACATCGTTT	35	Gammaproteobacteria	[61]
**PLA886^b)^**	GCCTTGCGACCATACTCCC	35	Planctomycetes	[62]
**HGC96a**	TATAGTTACCACCGCCGT	25	Actinomycetes	[63]
**CF319a**	TGGTCCGTGTCTCAGTAC	35	Bacteroidetes	[64]

^a) ^Probes were labelled with cy 3 ^b) ^For these probes, a competitor probe was used

### Statistical analyses

Data of FISH analysis we re arcsin transformed. For planctomycetes, data were additionally x^1/4 ^transformed to ensure equal variances. Plant species-, plant part-, and habitat-specific differences were analysed by 3-way ANOVAs (Sigma STAT 3.0). Non-metric dimensional scaling plots were generated with square-root transformation of data and Bray-Curtis similarity (Primer 5.0). For correlations, the Pearson correlation was used (Sigma STAT 3.0).

## Authors' contributions

MH: Guidance of experiments; writing the manuscript

MB: Carried out sampling and experiments

IB: Initiated the work on biofilm on charophytes together with MB, critically amended and edited the manuscript

EMG: Sampling in Lake Constance, supervising experiments, writing and editing the manuscript

All authors read and approved the final manuscript.
